# Supplementary material for: New Tricks with Old Dogs: Computational Identification and Experimental Validation of New miRNA–mRNA Regulation in hiPSC-CMs
Source: Biomedicines. 2022 Feb 6;10(2):391. doi: 10.3390/biomedicines10020391 (PMC8962266; doi:10.3390/biomedicines10020391)
Supplement: Supplementary file 1 [file biomedicines-10-00391-s001.zip › biomedicines-1511417-supplementary Figures.pdf]

Supplementary Figure S1

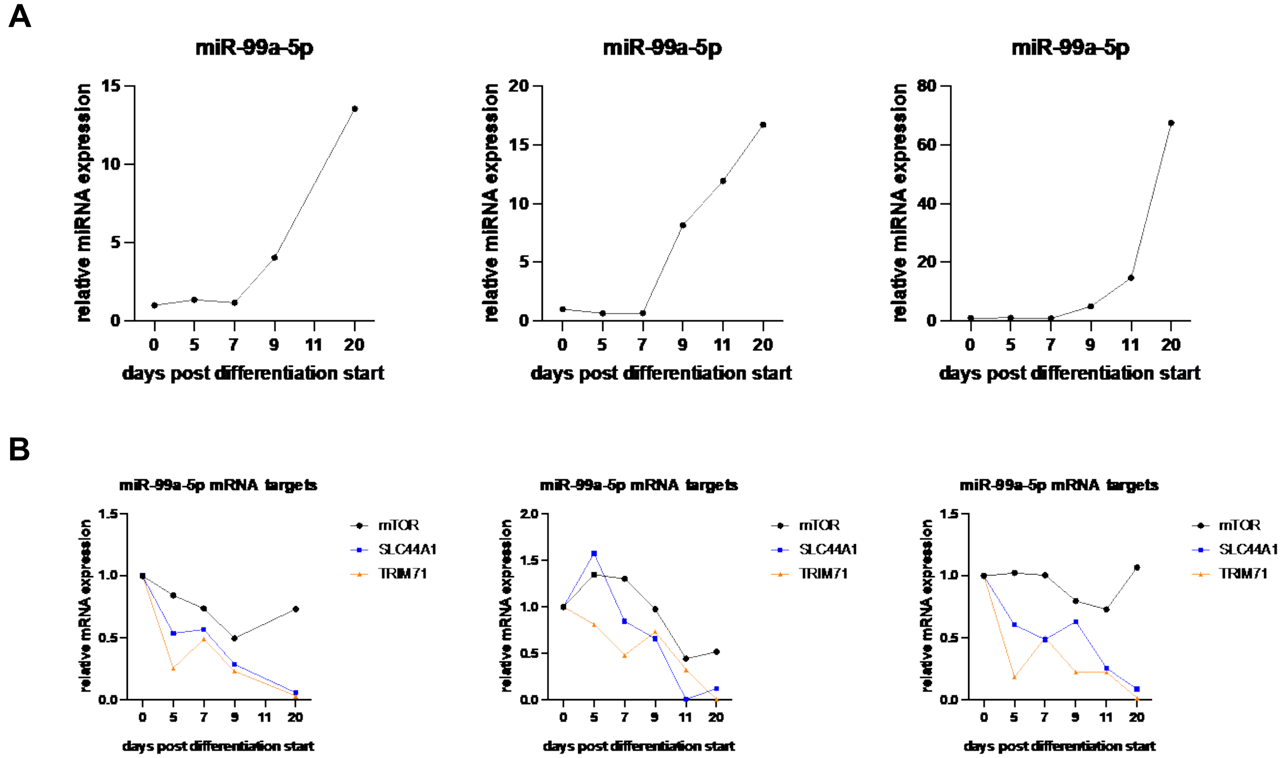

Supplementary Figure S1: Changing miRNA and mRNA expression levels in differentiating hiPSCs.

A. The diagrams show the changing expression levels of miR-99a-5p during hiPSC differentiation into cardiomyocytes in three independent experiments. RNA samples were collected on days 0, 5, 7, 9, 11 and 20 post differentiation start and miR-99a-5p levels were measured by real-time qPCR (RT-qPCR).

B. The diagram shows the changing expression levels of miR-99a-5p mRNA target transcripts during hiPSC differentiation into cardiomyocytes in three independent experiments. Experimental settings were as in A.

Supplementary Figure S2

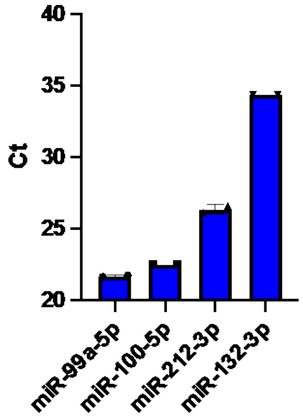

Supplementary Figure S2: Ct values for different miRNAs measured in 28 days old hiPSC-CMs.
